# Supplementary material for: The interactome of intact mitochondria by cross-linking mass spectrometry provides evidence for coexisting respiratory supercomplexes
Source: Mol Cell Proteomics. 2017 Dec 8;17(2):216–32. doi: 10.1074/mcp.RA117.000470 (PMC5795388; doi:10.1074/mcp.RA117.000470)
Supplement: Supplemental Data [file supp_RA117.000470_133922_0_supp_23978_fzffwf.zip › spectra_annotation/mito_DR_spectra_annotation/444-1-5-1-8-1.pdf]

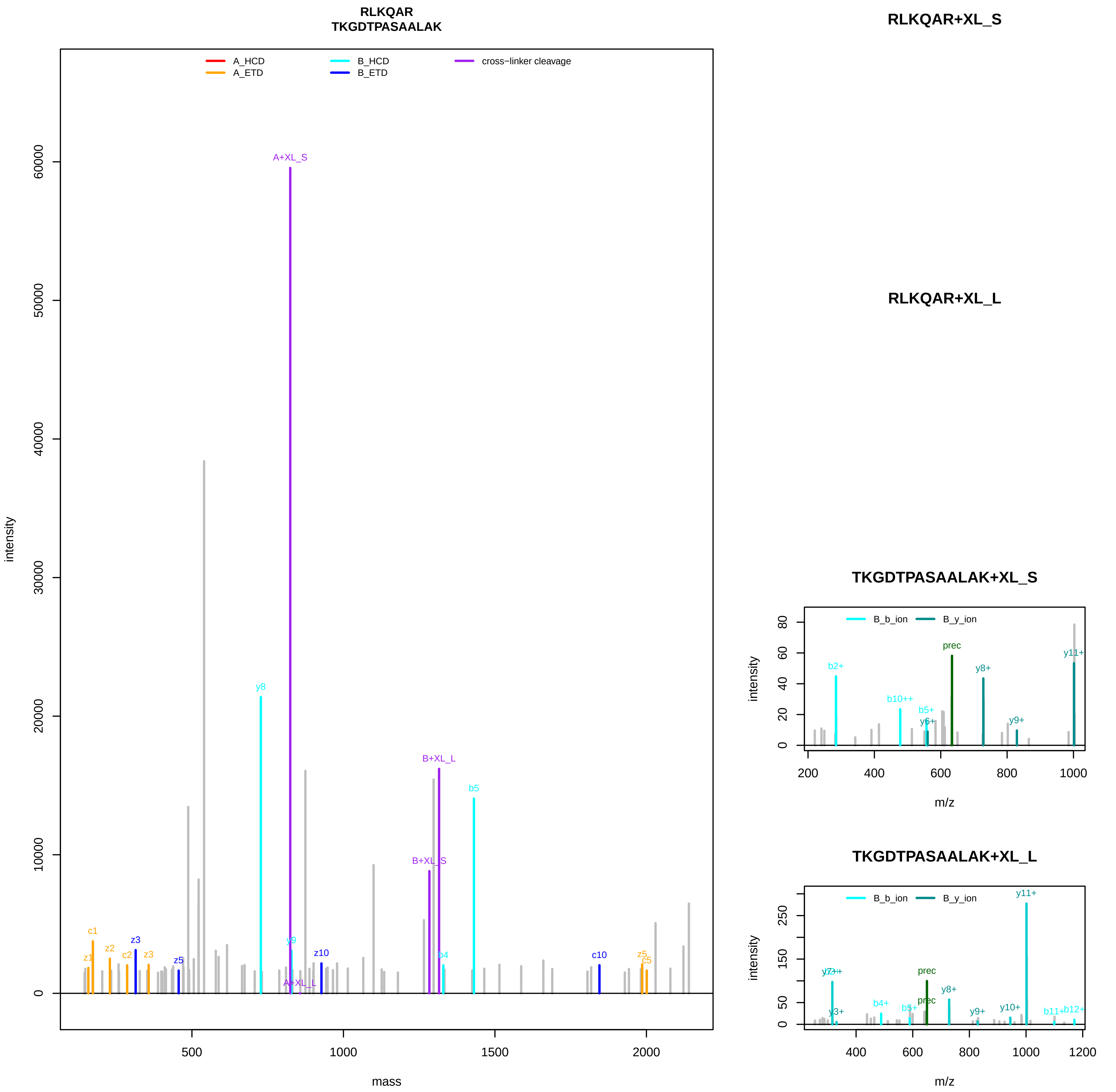
RLKQAR
TKGDTPASAALAK
A_HCD
A_ETD
B_HCD
B_ETD
cross−linker cleavage
A+XL_S
A+XL_L
B+XL_L
B+XL_S
y8
y9
b4
b5
z1
c1
z2
c2
z3
z3
z5
z10
c10
z5
c5
intensity
mass
0
10000
20000
30000
40000
50000
60000
500
1000
1500
2000
RLKQAR+XL_S
RLKQAR+XL_L
TKGDTPASAALAK+XL_S
B_b_ion
B_y_ion
prec
b2+
b10++
b5+
y6+
y8+
y9+
y11+
intensity
m/z
0
20
40
60
80
200
400
600
800
1000
TKGDTPASAALAK+XL_L
B_b_ion
B_y_ion
y11+
y7++
y3+
b4+
b5+
prec
y8+
y9+
y10+
b11+
b12+
intensity
m/z
0
50
150
250
400
600
800
1000
1200
